# Mortality of Philadelphia, for April, May and June, 1853, Arranged from the Record Kept at the Health Office

**Published:** 1853-08

**Authors:** Wilson Jewell


*Mortality of Philadelphia, for April, May and June,* 1853, *arranged from the record kept at the Health Office.* By WILSON JEWELL, M. D.

The deaths from all causes reported during the quarter ending July, 2d, embracing a period of 91 days, amount to 2235. Compared with those for the same period of last year, there has been a falling off of 15 per cent. in the aggregate.

Deducting the "Still Born," amounting to 150, the deaths from "External Causes," 82, "Old Age," 29, and "Unknown," 46, amounting in all to 307, and there will remain 1928 deaths from known diseases.

The number of deaths reported under one year of age, exclusive of still-born children, amounts to 482, or nearly one-fourth; and those under five years amount to 915, or about one half of the whole number of deaths from known diseases.

The deaths among males were 1157, females 1078—being an excess of 79 of the former over those of the latter.

Estimating the population at about 415,000, the aggregate of deaths in the quarter will give 1 in every 181½ of the population; which is equivalent to 25 deaths per day, during the quarter.

Eleven hundred and thirty-two, or about fifty per cent. of the whole number of deaths recorded, were under 20 years of age.

CLASS 1st.—Among this class, the "Endemic or Zymotic," there is a decrease of 162, or about 25 per cent. from those for the like period in 1852. The difference is accounted for by the falling off in small-pox and measles. Last year the mortality from these diseases was wide-spread. The present year, thus far, they are comparatively rare.

CLASS 2d.—Of the diseases of "Uncertain Seat," there were 297 deaths. Of these, 212 are charged to Debility, Marasmus and Dropsy, of which number 103 were under one year of age.

CLASS 3d.—The deaths under this head, "Nervous System," amount to 393. 142, or nearly one-third, were from Convulsions, 47 from Dropsy of the Brain, and 40 from Inflammation of the Brain. Of these, 114 were under one year of age.

CLASS 4th.—The diseases of the "Organs of Respiration," amounting to 515, constitute nearly one-fourth of the whole number of deaths for the quarter.

Consumption of the Lungs make up a large proportion of this class—namely, 312; while Inflammation of the Lungs furnish 103. The deaths from Consumption of the Lungs are less 12 than for the same period in 1852. 92 of the deaths took place between the ages of 20 and 30. The deaths among females exceed those of males by 11 per cent.

CLASS 8th.—The diseases of the "Organs of Generation" furnish only 22 deaths. Five of these are charged to Child-bed and four to Puerperal Fever. In 1852, in the same period, there were 24 deaths from diseases of the Organs of Generation, eight of which were from Puerperal Fever.

CLASS 12th.—From "External Causes" there were 82 deaths. 56 of these occurred in males and 26 among females. The exposed situation of the male sex over that of females, and the danger of their out-door employments, may add to the increase of deaths among males from external causes.

Table No. 3, furnishes the exact number of deaths at fifteen distinct periods of life. By this it will be seen that infancy furnishes a large proportion of the deaths; that between birth and the fifth year, 915 children have perished during the past three months—a number almost equal to one-half the whole number at every age of life. The healthiest period of childhood lies between the ages of 10 and 15, and this corresponds with other statistics.

The deaths that took place at the Alms-House during the quarter, were 174, and those which occurred among people of color were 190.

### TABLE No. I.

*Deaths for the Second Quarter of* 1853, *classified.*

| | April. | May. | June. | MALE. A. | MALE. M. | MALE. J. | FEMALE. A. | FEMALE. M. | FEMALE. J. | Total. |
|---|---|---|---|---|---|---|---|---|---|---|
| 1. *Endemic and Contagious Diseases.* Zymotic or Epidemic . . | 135 | 108 | 218 | 59 | 49 | 112 | 76 | 59 | 106 | 461 |
| 2. *Uncertain or general seat.* Sporadic diseases . . . . | 94 | 76 | 127 | 55 | 36 | 63 | 39 | 40 | 64 | 297 |
| 3. Nervous System . . . | 140 | 104 | 149 | 77 | 52 | 89 | 63 | 52 | 60 | 393 |
| 4. Organs of Respiration . . . | 203 | 152 | 160 | 99 | 83 | 78 | 104 | 69 | 82 | 515 |
| 5. " Circulation . . . | 24 | 17 | 23 | 12 | 6 | 8 | 12 | 11 | 15 | 64 |
| 6. Digestive Organs . . . | 54 | 41 | 56 | 31 | 16 | 33 | 23 | 25 | 23 | 151 |
| 7. Urinary Organs . . . | 1 | 2 | 6 | 1 | 2 | 5 | | | 1 | 9 |
| 8. Organs of Generation . . | 6 | 11 | 5 | | | | 6 | 11 | 5 | 22 |
| 9. " Locomotion . . | 6 | 5 | 1 | 1 | 2 | | 5 | 3 | 1 | 12 |
| 10. Integumentary System . . | 1 | 2 | 1 | 1 | 1 | 1 | | 1 | | 4 |
| 11. Old Age . . . . . . | 14 | 6 | 9 | 6 | 3 | 6 | 8 | 3 | 3 | 29 |
| 12. External Causes . . . . . | 19 | 26 | 37 | 11 | 17 | 28 | 8 | 9 | 9 | 82 |
| Still Born . . . . . . | 50 | 33 | 67 | 31 | 21 | 36 | 19 | 12 | 31 | 150 |
| Unknown . . . . . . | 9 | 14 | 23 | 6 | 5 | 15 | 3 | 9 | 8 | 46 |
| | 756 | 597 | 882 | 390 | 293 | 474 | 366 | 304 | 408 | 2235 |

### TABLE No. II.

1. *Endemic and Contagious Diseases.—Zymotic or Epidemic.*

| | Male. | Female. | Under 1 yr. | 1 to 2. | 2 to 5. | 5 to 10. | 10 to 15. | 15 to 20. | 20 to 30. | 30 to 40. | 40 to 50. | 50 to 60. | 60 to 70. | 70 to 80. | 80 to 90. | 90 to 100. | 100 to 110. | Total. |
|---|---|---|---|---|---|---|---|---|---|---|---|---|---|---|---|---|---|---|
| Cholera Infantum . | 28 | 35 | 53 | 8 | 2 | | | | | | | | | | | | | 63 |
| " Morbus . | 2 | 2 | 2 | | | | | | 1 | | | 1 | | | | | | 4 |
| Croup . . . | 25 | 29 | 7 | 8 | 30 | 9 | | | | | | | | | | | | 54 |
| Diarrhœa . . . | 17 | 12 | 14 | 3 | 1 | | | | 4 | 2 | 1 | | 1 | | 3 | | | 29 |
| Dysentery . . | 31 | 31 | 18 | 12 | 15 | 1 | | 1 | | 2 | 2 | 2 | 5 | 1 | 2 | 1 | | 62 |
| Erysipelas . . | 14 | 12 | 10 | 1 | 2 | | 1 | 1 | 2 | 2 | 1 | 3 | 2 | | 1 | | | 26 |
| Fever . . . | 2 | 4 | 1 | | | | | | 1 | 1 | 1 | 1 | 1 | | | | | 6 |
| " Bilious . . | | 4 | | 1 | 1 | | | 1 | | | 1 | | | | | | | 4 |
| " Continued . | | 3 | | | | | | | | 2 | | 1 | | | | | | 3 |
| " Intermittent . | | 3 | | | 1 | | | | | 2 | | | | | | | | 3 |
| " Remittent . | 5 | 7 | | 3 | | | | 2 | 2 | 3 | | 1 | | 1 | | | | 12 |
| " Scarlet . . | 37 | 38 | 4 | 13 | 34 | 19 | 4 | 1 | | | | | | | | | | 75 |
| " Typhus . . | 26 | 23 | | | | 1 | 1 | 4 | 12 | 12 | 6 | 8 | 2 | 2 | 1 | | | 49 |
| " Typhoid . . | 19 | 23 | | | 1 | 5 | 4 | 3 | 6 | 5 | 7 | 7 | 1 | 1 | 1 | 1 | | 42 |
| Hooping Cough . . | 3 | 4 | 2 | 5 | | | | | | | | | | | | | | 7 |
| Influenza . . . | | 1 | | 1 | | | | | | | | | | | | | | 1 |
| Measles . . . . | 3 | 2 | 1 | 1 | 3 | | | | | | | | | | | | | 5 |
| Small Pox. . . | 7 | 8 | 7 | 1 | 2 | 2 | | 1 | 1 | 1 | | | | | | | | 15 |
| Syphilis . . . | 1 | | | | | | | | | 1 | | | | | | | | 1 |
| | 220 | 241 | 119 | 57 | 92 | 37 | 10 | 14 | 29 | 33 | 19 | 24 | 12 | 5 | 8 | 2 | | 461 |

### 2. *Uncertain or General Seat.—Sporadic Diseases.*

| | Male. | Female. | Under 1 yr. | 1 to 2. | 2 to 5. | 5 to 10. | 10 to 15. | 15 to 20. | 20 to 30. | 30 to 40. | 40 to 50. | 50 to 60. | 60 to 70. | 70 to 80. | 80 to 90. | 90 to 100. | 100 to 110. | Total. |
|---|---|---|---|---|---|---|---|---|---|---|---|---|---|---|---|---|---|---|
| Abscess . . . | 4 | | | 1 | | | | 1 | 1 | 1 | | | | | | | | 4 |
| " Neck . . | | 1 | | 1 | | | | | | | | | | | | | | 1 |
| " Psoas . . | 1 | 1 | | | | | | | | | | 1 | 1 | | | | | 2 |
| Angina . . . | | 4 | 2 | | | 2 | | | | | | | | | | | | 4 |
| Cancer . . . | 2 | 3 | | | | | 1 | | | | | 4 | | | | | | 5 |
| " Breast . . | | 1 | | | | | | | | | | 1 | | | | | | 1 |
| Cachexia . . . | 1 | 1 | | | 1 | | | | | | | | 1 | | | | | 2 |
| Cyanosis . . . | 2 | 3 | 5 | | | | | | | | | | | | | | | 5 |
| Debility . . . | 45 | 50 | 40 | 3 | 2 | | 1 | 1 | 3 | 9 | 3 | 7 | 11 | 9 | 6 | | | 95 |
| Disease of Throat . | 1 | | | | | | | | | | 1 | | | | | | | 1 |
| Dropsy . . . | 15 | 11 | 4 | | 5 | 2 | | 1 | 3 | 1 | 1 | 1 | 4 | 2 | 1 | 1 | | 26 |
| Fungous Glands of Neck | 1 | | | | | | | | | | | | 1 | | | | | 1 |
| Gangrene . . . | | 2 | | 1 | 1 | | | | | | | | | | | | | 2 |
| Glandular Swelling . | | 1 | 1 | | | | | | | | | | | | | | | 1 |
| Hectic Fever . . | 2 | | | | | | | | 1 | 1 | | | | | | | | 2 |
| Hemorrhage . . | 3 | 5 | 4 | | 2 | | | | 1 | | | | 1 | | | | | 8 |
| Inanition . . . | 5 | 3 | 5 | | 1 | | | | | | 1 | 1 | | | | | | 8 |
| Inflammation . . | 3 | | | | 2 | | | | | | 1 | | | | | | | 3 |
| " Throat . | | 1 | | | 1 | | | | | | | | | | | | | 1 |
| " Tonsils . | 1 | | | 1 | | | | | | | | | | | | | | 1 |
| Malformation . . | 1 | 3 | 3 | 1 | | | | | | | | | | | | | | 4 |
| Marasmus . . . | 44 | 47 | 59 | 11 | 14 | | 2 | 1 | | 1 | 3 | | | | | | | 91 |
| Melœna . . . | 1 | 1 | 1 | | | | | | | | 1 | | | | | | | 2 |
| Scirrhus . . . | 1 | | | | | | | | | | | 1 | | | | | | 1 |
| Scrofula . . . | 9 | 3 | 6 | 1 | 1 | 1 | | 1 | | 1 | | | 1 | | | | | 12 |
| Spina Bifida . . | 1 | 1 | 2 | | | | | | | | | | | | | | | 2 |
| Tabes Mesenterica . | 7 | 1 | 6 | 1 | 1 | | | | | | | | | | | | | 8 |
| Ulceration . . . | 2 | | | | | | | | | 1 | | 1 | | | | | | 2 |
| " Throat . | 2 | | | 1 | | | | | | 1 | | | | | | | | 2 |
| | 154 | 143 | 138 | 22 | 31 | 5 | 4 | 5 | 9 | 16 | 11 | 17 | 20 | 11 | 7 | 1 | | 297 |

### 3. *Of the Nervous System.*

| | Male. | Female. | Under 1 yr. | 1 to 2. | 2 to 5. | 5 to 10. | 10 to 15. | 15 to 20. | 20 to 30. | 30 to 40. | 40 to 50. | 50 to 60. | 60 to 70. | 70 to 80. | 80 to 90. | 90 to 100. | 100 to 110. | Total. |
|---|---|---|---|---|---|---|---|---|---|---|---|---|---|---|---|---|---|---|
| Abscess of Brain . . | 1 | | | | | 1 | | | | | | | | | | | | 1 |
| Apoplexy . . . | 12 | 11 | | | 1 | | | 1 | 1 | 1 | 5 | 4 | 6 | 4 | | | | 23 |
| Cerebral Fever . . | | 1 | | | 1 | | | | | | | | | | | | | 1 |
| Compression of Brain . | 1 | | 1 | | | | | | | | | | | | | | | 1 |
| Concussion " . | 2 | 1 | | 2 | | | | | | 1 | | | | | | | | 3 |
| Congestion " . | 19 | 14 | 11 | 4 | 8 | | 1 | | 4 | 2 | 1 | 1 | | 1 | | | | 33 |
| Convulsions . . | 69 | 73 | 80 | 19 | 25 | 3 | 4 | 1 | 1 | 5 | 2 | 1 | 1 | | | | | 142 |
| " Puerperal . | | 1 | | | | | | | | 1 | | | | | | | | 1 |
| Coup de Soleil . . | 20 | 4 | | 1 | | 1 | | 3 | 7 | 5 | 5 | 1 | | | 1 | | | 24 |
| Cramp . . . | 1 | | 1 | | | | | | | | | | | | | | | 1 |
| Dropsy of Brain . . | 23 | 24 | 23 | 12 | 8 | 3 | | | | | | 1 | | | | | | 47 |
| Effusion " . . | 6 | 8 | 4 | 1 | 3 | 1 | | 1 | 1 | | | 1 | 2 | | | | | 14 |
| Epilepsy . . . | 3 | 3 | 1 | | | | | 2 | 3 | | | | | | | | | 6 |
| Neuralgia . . . | 1 | 1 | | | | | | | 1 | | | | 1 | | | | | 2 |
| Palsy . . . . | 6 | 7 | | | | | | | 1 | | 2 | 3 | 2 | | 5 | | | 13 |
| " of Brain . . | 1 | | | | 1 | | | | | | | | | | | | | 1 |
| Mania a potu . . | 19 | 1 | | | | | | 1 | 4 | 4 | 6 | 3 | 2 | | | | | 20 |
| Softening of Brain . | 1 | 1 | | | | | | | | | 1 | | | | 1 | | | 2 |
| Tetanus . . . | 3 | | 1 | | | | | | 1 | | 1 | | | | | | | 3 |
| Trismus . . . | 1 | 1 | 1 | 1 | | | | | | | | | | | | | | 2 |
| Tumors of Brain . | 1 | | | | | | | 1 | | | | | | | | | | 1 |
| Disease " . . | 6 | 6 | 2 | 1 | 2 | | | 1 | 1 | 2 | 3 | | | | | | | 12 |
| Inflammation " . : | 22 | 18 | 11 | 9 | 11 | 4 | 2 | | 1 | 1 | | 1 | | | | | | 40 |
| | 218 | 175 | 136 | 50 | 60 | 13 | 7 | 11 | 26 | 22 | 26 | 16 | 14 | 5 | 7 | | | 393 |

### 4. *Organs of Respiration.*

| | Male. | Female. | Under 1 yr. | 1 to 2. | 2 to 5. | 5 to 10. | 10 to 15. | 15 to 20. | 20 to 30. | 30 to 40. | 40 to 50. | 50 to 60. | 60 to 70. | 70 to 80. | 80 to 90. | 90 to 100. | 100 to 110. | Total. |
|---|---|---|---|---|---|---|---|---|---|---|---|---|---|---|---|---|---|---|
| Abscess of Lungs . | 1 | 3 | | | 1 | | 1 | | | | | 2 | | | | | | 4 |
| " Thorax . | | 1 | 1 | | | | | | | | | | | | | | | 1 |
| Asthma . . . | | 3 | | | | | | | | 1 | | | 2 | | | | | 3 |
| " Thymic . . | | 1 | | | | | 1 | | | | | | | | | | | 1 |
| Consumption of Lungs . | 147 | 165 | 3 | 4 | 3 | 5 | 9 | 30 | 92 | 74 | 50 | 20 | 16 | 6 | | | | 312 |
| Congestion " . | 4 | 5 | 3 | 3 | | | | | 1 | | | | 2 | | | | | 9 |
| Disease of Lungs . | 8 | 1 | 3 | 1 | | 1 | | | 3 | | 1 | | | | | | | 9 |
| Dropsy of Chest . . | 4 | 6 | | 1 | 2 | | 2 | 1 | | 1 | | | 1 | 2 | | | | 10 |
| Effusion " . . | 2 | 1 | | | 1 | 2 | | | | | | | | | | | | 3 |
| Empyema . . . | 1 | | | | | | | | 1 | | | | | | | | | 1 |
| Disease of Chest . . | 1 | | | | | 1 | | | | | | | | | | | | 1 |
| Effusion of Lungs . | | 1 | | | | | | | | | | | | 1 | | | | 1 |
| Hemorrhage of Lungs . | | 4 | 1 | | | | | | | 1 | 1 | | 1 | | | | | 4 |
| Inflammation " . | 61 | 42 | 16 | 9 | 20 | 5 | 1 | 2 | 14 | 11 | 5 | 7 | 5 | 7 | 1 | | | 103 |
| " Larynx . | 5 | 1 | 2 | | 1 | | | 1 | 1 | 1 | | | | | | | | 6 |
| " Bronchiæ . | 19 | 16 | 13 | 4 | 5 | | | | 2 | 1 | 1 | 3 | 3 | 3 | | | | 35 |
| " Pleura . | 2 | 2 | | | | | | | 3 | | | 1 | | | | | | 4 |
| " Chest . | 2 | 1 | | 2 | 1 | | | | | | | | | | | | | 3 |
| Palsy of Lungs . . | 1 | | | | 1 | | | | | | | | | | | | | 1 |
| Ulceration of Larynx . | 1 | 1 | | 1 | | | | | 1 | | | | | | | | | 2 |
| Tuberculosis . . | 1 | 1 | | | | | 1 | | | | | 1 | | | | | | 2 |
| | 260 | 255 | 42 | 25 | 35 | 14 | 15 | 34 | 118 | 90 | 58 | 34 | 30 | 19 | 1 | | | 515 |

5. *Organs of Circulation.*

| | Male. | Female. | Under 1 yr. | 1 to 2. | 2 to 5. | 5 to 10. | 10 to 15. | 15 to 20. | 20 to 30. | 30 to 40. | 40 to 50. | 50 to 60. | 60 to 70. | 70 to 80. | 80 to 90. | 90 to 100. | 100 to 110. | Total. |
|---|---|---|---|---|---|---|---|---|---|---|---|---|---|---|---|---|---|---|
| Angina Pectoris . . | | 1 | | | | | | | | | | 1 | | | | | | 1 |
| Anæmia . . . | 1 | 1 | | | 1 | | | | | | 1 | | | | | | | 2 |
| Dropsy of Heart . . | 3 | 6 | 2 | | 1 | 1 | 1 | | | 1 | 1 | | 1 | | | 1 | | 9 |
| Disease " . . | 17 | 23 | 4 | 4 | 3 | 1 | 2 | 1 | 2 | 6 | 6 | 5 | 2 | 2 | 2 | | | 40 |
| Enlargem't " . . | 1 | 5 | | 1 | | | | 1 | 1 | | 1 | 1 | | 1 | | | | 6 |
| Inflammation " . . | 4 | 1 | | | | | | 1 | 1 | | 2 | 1 | | | | | | 5 |
| Ossification " . . | | 1 | | | | | | | | | | | | 1 | | | | 1 |
| | 26 | 38 | 6 | 5 | 5 | 2 | 3 | 3 | 4 | 7 | 11 | 8 | 3 | 4 | 2 | 1 | | 64 |

6. *Digestive Organs.*

| | Male. | Female. | Under 1 yr. | 1 to 2. | 2 to 5. | 5 to 10. | 10 to 15. | 15 to 20. | 20 to 30. | 30 to 40. | 40 to 50. | 50 to 60. | 60 to 70. | 70 to 80. | 80 to 90. | 90 to 100. | 100 to 110. | Total. |
|---|---|---|---|---|---|---|---|---|---|---|---|---|---|---|---|---|---|---|
| Abscess of Liver . | 1 | 1 | | | | | | | 1 | 1 | | | | | | | | 2 |
| Abdominal Dropsy . | 3 | 3 | | | | | | | 2 | 1 | | 1 | 1 | 1 | | | | 6 |
| Cancer of Stomach . | 1 | | | | | | | | | | 1 | | | | | | | 1 |
| Cancrum Oris . . | 3 | 2 | | 1 | 2 | 1 | 1 | | | | | | | | | | | 5 |
| Cirrhosis . . . | 1 | 1 | | | | | | | | | | | 2 | | | | | 2 |
| Consumption of Bowels | 1 | 1 | | | | 1 | | | 1 | | | | | | | | | 2 |
| Disease of Bowels . | 1 | 1 | | | 1 | | | | | | | 1 | | | | | | 2 |
| " Liver . . | 2 | 1 | 1 | | | | | | | 1 | 1 | | | | | | | 3 |
| " Stomach . | | 2 | | | | | | | 1 | 1 | | | | | | | | 2 |
| Dyspepsia . . . | 1 | | | 1 | | | | | | | | | | | | | | 1 |
| Enlargement of Liver . | 1 | | | | | | | | 1 | | | | | | | | | 1 |
| Gastric Fever . . | 1 | | | 1 | | | | | | | | | | | | | | 1 |
| Hernia, Strangulated . | 1 | | 1 | | | | | | | | | | | | | | | 1 |
| Intussusception . . | 1 | | | | 1 | | | | | | | | | | | | | 1 |
| Inflammation of Bowels | 2 | 2 | 2 | | | 2 | | | | | | | | | | | | 4 |
| " Liver . . | 7 | 3 | | | 2 | | 1 | 1 | | 2 | 2 | 1 | 1 | | | | | 10 |
| " Stom'h & Bow'ls | 32 | 29 | 12 | 12 | 10 | 1 | | 3 | 9 | 3 | 5 | 2 | 1 | 1 | 1 | 1 | | 61 |
| " Peritoneum . | 10 | 13 | 1 | | 2 | | 2 | 2 | 6 | 5 | 1 | 3 | 1 | | | | | 23 |
| Jaundice . . . | 5 | 2 | 3 | | 1 | 1 | | | 1 | | | | 1 | | | | | 7 |
| Obstruction of Bowels . | 1 | 1 | | | | | | | | 1 | | | 1 | | | | | 2 |
| Perforation of Stomach | 1 | | | | | | | | | 1 | | | | | | | | 1 |
| Scirrhus of Pancreas . | | 1 | | | | | | | | | | 1 | | | | | | 1 |
| " of Stomach . | 2 | | | | | | | | | 1 | 1 | | | | | | | 2 |
| " of Bowels . | | 1 | | | | | | | | | | 1 | | | | | | 1 |
| Teething . . . | 2 | 3 | 3 | 2 | | | | | | | | | | | | | | 5 |
| Tumour of Abdomen . | 1 | | | | | | | | | | | | | 1 | | | | 1 |
| Worms . . . | | 1 | | | | 1 | | | | | | | | | | | | 1 |
| Ulceration of Bowels . | | 2 | | | | | | | | 1 | | | 1 | | | | | 2 |
| | 81 | 70 | 23 | 17 | 19 | 7 | 4 | 6 | 22 | 18 | 11 | 10 | 9 | 3 | 1 | 1 | | 151 |

7. *The Urinary Organs.*

| | Male. | Female. | Under 1 yr. | 1 to 2. | 2 to 5. | 5 to 10. | 10 to 15. | 15 to 20. | 20 to 30. | 30 to 40. | 40 to 50. | 50 to 60. | 60 to 70. | 70 to 80. | 80 to 90. | 90 to 100. | 100 to 110. | Total. |
|---|---|---|---|---|---|---|---|---|---|---|---|---|---|---|---|---|---|---|
| Albuminaria . . | 1 | | | | | | | | 1 | | | | | | | | | 1 |
| Disease of Bladder . | 1 | | | | | | | | | | | | | 1 | | | | 1 |
| Inflam. of Bladder . | 1 | 1 | | | | | | | 1 | | | | 1 | | | | | 2 |
| " of Kidneys . | 1 | | | | 1 | | | | | | | | | | | | | 1 |
| Ischuria . . . | 1 | | | | | | | | 1 | | | | | | | | | 1 |
| Rupture of Urethra . | 1 | | | | | | | | | 1 | | | | | | | | 1 |
| Stricture . . . | 1 | | | | | | | | | | | | 1 | | | | | 1 |
| Urinary Calculi . . | 1 | | | | | | | | | | | | 1 | | | | | 1 |
| | 8 | 1 | | | 1 | | | | 3 | 1 | | | 3 | 1 | | | | 9 |

8. *Organs of Generation.*

| | Male. | Female. | Under 1 yr. | 1 to 2. | 2 to 5. | 5 to 10. | 10 to 15. | 15 to 20. | 20 to 30. | 30 to 40. | 40 to 50. | 50 to 60. | 60 to 70. | 70 to 80. | 80 to 90. | 90 to 100. | 100 to 110. | Total. |
|---|---|---|---|---|---|---|---|---|---|---|---|---|---|---|---|---|---|---|
| Cancer of Uterus . . | | 3 | | | | | | | | 1 | 1 | | 1 | | | | | 3 |
| Child Bed . . . | | 5 | | | | | | | 1 | 4 | | | | | | | | 5 |
| Disease of Uterus . | | 1 | | | | | | | | | | | 1 | | | | | 1 |
| Fever Puerperal . . | | 4 | | | | | | | 3 | 1 | | | | | | | | 4 |
| Hemorrhage from Uterus | | 3 | | | | | | | 2 | 1 | | | | | | | | 3 |
| Inflammation of " | | 3 | | | | | | | | 2 | 1 | | | | | | | 3 |
| Puerperal Mania . | | 2 | | | | | | | 2 | | | | | | | | | 2 |
| Scirrhus of Uterus . | | 1 | | | | | | | | | | | 1 | | | | | 1 |
| | | 32 | | | | | | | 8 | 9 | 2 | | 3 | | | | | 22 |

9. *Organs of Locomotion.*

| | Male. | Female. | Under 1 yr. | 1 to 2. | 2 to 5. | 5 to 10. | 10 to 15. | 15 to 20. | 20 to 30. | 30 to 40. | 40 to 50. | 50 to 60. | 60 to 70. | 70 to 80. | 80 to 90. | 90 to 100. | 100 to 110. | Total. |
|---|---|---|---|---|---|---|---|---|---|---|---|---|---|---|---|---|---|---|
| Caries . . . . | | 2 | | | | | | | | | | | 1 | | 1 | | | 2 |
| Caries of Spine . . | 1 | 1 | | | | | 1 | 1 | | | | | | | | | | 2 |
| Congestion Spinal Marrow | | 1 | | | | | | | | | | | 1 | | | | | 1 |
| Disease of Hip . . | | 1 | | | | 1 | | | | | | | | | | | | 1 |
| " of Spine . . | 2 | 1 | | 1 | | 1 | 1 | | | | | | | | | | | 3 |
| Inflam. of Knee Joint . | | 1 | | | | | | | | | | | 1 | | | | | 1 |
| " of Spine . . | | 1 | | | | 1 | | | | | | | | | | | | 1 |
| Spinal Irritation . . | | 1 | | | | 1 | | | | | | | | | | | | 1 |
| | 3 | 9 | | 1 | | 4 | 2 | 1 | | | | | 3 | | 1 | | | 12 |

### 10. *The Integumentary System.*

| | Male. | Female. | Under 1 yr. | 1 to 2. | 2 to 5. | 5 to 10. | 10 to 15. | 15 to 20. | 20 to 30. | 30 to 40. | 40 to 50. | 50 to 60. | 60 to 70. | 70 to 80. | 80 to 90. | 90 to 100. | 100 to 110. | Total. |
|---|---|---|---|---|---|---|---|---|---|---|---|---|---|---|---|---|---|---|
| Carbuncle . . . | | 1 | | | | | | | | 1 | | | | | | | | 1 |
| Fistula . . . | 1 | | | | | | | | 1 | | | | | | | | | 1 |
| Purpura Hemorrhagica | 2 | | 1 | | | | | | 1 | | | | | | | | | 2 |
| | 3 | 1 | 1 | | | | | | 2 | 1 | | | | | | | | 4 |

### 11. *Old Age.*

| | Male. | Female. | Under 1 yr. | 1 to 2. | 2 to 5. | 5 to 10. | 10 to 15. | 15 to 20. | 20 to 30. | 30 to 40. | 40 to 50. | 50 to 60. | 60 to 70. | 70 to 80. | 80 to 90. | 90 to 100. | 100 to 110. | Total. |
|---|---|---|---|---|---|---|---|---|---|---|---|---|---|---|---|---|---|---|
| Old Age . . . | 15 | 14 | | | | | | | | | | | 3 | 11 | 14 | 1 | | 29 |

### 12. *From External Causes.*

| | Male. | Female. | Under 1 yr. | 1 to 2. | 2 to 5. | 5 to 10. | 10 to 15. | 15 to 20. | 20 to 30. | 30 to 40. | 40 to 50. | 50 to 60. | 60 to 70. | 70 to 80. | 80 to 90. | 90 to 100. | 100 to 110. | Total. |
|---|---|---|---|---|---|---|---|---|---|---|---|---|---|---|---|---|---|---|
| Asphyxia . . . | 2 | 2 | 4 | | | | | | | | | | | | | | | 4 |
| Burns and Scalds . . | 3 | 7 | | 4 | | 1 | | | 2 | 3 | | | | | | | | 10 |
| Casualties . . . | 19 | 10 | 4 | | 2 | 2 | 2 | 2 | 3 | 3 | 8 | 1 | 1 | 1 | | | | 29 |
| Drowned . . . | 20 | 1 | | | 1 | 2 | 2 | 2 | 4 | 2 | 4 | 1 | 2 | 1 | | | | 21 |
| Fracture . . . | 1 | | | | | | | | 1 | | | | | | | | | 1 |
| " of Spine . | 1 | | | | | | | | | | | 1 | | | | | | 1 |
| Intemperance . . | 5 | 3 | | | | | | | 3 | 2 | 1 | 1 | 1 | | | | | 8 |
| Poisoning . . . | | 2 | 1 | | | | | 1 | | | | | | | | | | 2 |
| Suffocation . . . | 1 | | | | | | | | | 1 | | | | | | | | 1 |
| Suicide . . . | 4 | 1 | | | | | | | 1 | 1 | 2 | 1 | | | | | | 5 |
| | 56 | 26 | 9 | 4 | 3 | 5 | 4 | 5 | 14 | 12 | 15 | 5 | 4 | 2 | | | | 82 |
| Unknown . . . | 26 | 20 | 8 | 3 | 3 | 1 | 1 | | 6 | 6 | 5 | 6 | 5 | 2 | | | | 46 |
| Still Born . . . | 88 | 62 | 150 | | | | | | | | | | | | | | | 150 |

### TABLE NO. 3.

*Deaths for the Second Quarter of* 1853, *at fifteen distinct periods of life.*

| | |
|---|---|
| Under 1 year, . . . . . . | 482 |
| 1 to 2 . . . . . . . | 184 |
| 2 to 5 . . . . . . . | 249 |
| 5 to 10 . . . . . . . | 88 |
| 10 to 15 . . . . . . . | 50 |
| 15 to 20 . . . . . . . | 79 |
| 20 to 0 . . . . . . . | 241 |
| 30 to 40 . . . . . . . | 215 |
| 40 to 50 . . . . . . . | 158 |
| 50 to 60 . . . . . . . | 120 |
| 60 to 70 . . . . . . . | 109 |
| 70 to 80 . . . . . . . | 63 |
| 80 to 90 . . . . . . . | 41 |
| 90 to 100 . . . . . . . | 6 |
| 100 to 110 . . . . . . . | 0 |
| | 2085 |
| Still Born . . . . . . . | 150 |
| Total, | 2235 |

Included in the above Tables, are 174 from the Blockley Almshouse, 190 people of color, and 20 from the country; as follows:

| | April. | May. | June. | Total. |
|---|---|---|---|---|
| Almshouse, . . | 65 | 47 | 62 | 174 |
| Blacks, . . . | 67 | 55 | 68 | 190 |
| Country, . . | 7 | 5 | 8 | 20 |
| | 139 | 107 | 138 | 384 |

